# LINC00152 expression in normal and Chronic Lymphocytic Leukemia B cells

**DOI:** 10.1002/hon.2938

**Published:** 2021-10-28

**Authors:** Serena Matis, Martina Rossi, Lorenzo Brondolo, Martina Cardillo, Daniele Reverberi, Rosanna Massara, Monica Colombo, Adalberto Ibatici, Emanuele Angelucci, Tiziana Vaisitti, Silvia Bruno, Sonia Fabris, Antonino Neri, Massimo Gentile, Fortunato Morabito, Giovanna Cutrona, Paola Briata, Roberto Gherzi, Franco Fais

**Affiliations:** ^1^ Molecular Pathology Unit IRCCS Ospedale Policlinico San Martino Genoa Italy; ^2^ Gene Expression Regulation Laboratory IRCCS Ospedale Policlinico San Martino Genoa Italy; ^3^ Hematology Unit and Transplant Center IRCCS Ospedale Policlinico San Martino Genoa Italy; ^4^ Department of Medical Sciences University of Torino Torino Italy; ^5^ Department of Experimental Medicine University of Genoa Genoa Italy; ^6^ Fondazione Cà Granda IRCCS Hematology Ospedale Maggiore Policlinico Milano Milan Italy; ^7^ Department of Oncology and Hemato‐oncology University of Milan Milan Italy; ^8^ Hematology Unit A.O. of Cosenza Cosenza Italy; ^9^ Biotechnology Research Unit AO Cosenza Italy; ^10^ Hematology and Bone Marrow Transplant Unit, Hemato‐Oncology Department Augusta Victoria Hospital East Jerusalem Israel

**Keywords:** chronic lymphocytic leukemia, LINC00152, long non‐coding RNAs, memory B cells, naïve B cells

## Abstract

Long non‐coding RNAs are emerging as essential regulators of gene expression, but their role in normal and neoplastic B cells is still largely uncharacterized. Here, we report on the expression pattern of the LINC00152 in normal B cells and Chronic Lymphocytic Leukemia B cell clones. Higher LINC00152 levels were consistently observed in memory B cell populations when compared to naïve B cells in the normal tissues analyzed [peripheral blood (PB), tonsils, and spleen]. In addition, independent stimulation via Immunoglobulins (IG), CD40, or Toll‐like Receptor 9 (TLR9) upregulated LINC00152 in PB B cells. The expression of LINC00152 in a cohort of 107 early stage Binet A CLL patients was highly variable and did not correlate with known prognostic markers or clinical evolution. TLR9 stimulation, but not CD40 or IG challenge, was able to upregulate LINC00152 expression in CLL cells. In addition, LINC00152 silencing in CLL cell lines expressing LINC00152 failed to induce significant cell survival or apoptosis changes. These data suggest that, in normal B cells, the expression of LINC00152 is regulated by immunomodulatory signals, which are only partially effective in CLL cells. However, LINC00152 does not appear to contribute to CLL cell expansion and/or survival in a cohort of newly diagnosed CLL patients.

## 1 INTRODUCTION

Non‐coding RNAs (ncRNAs) constitute more than 90% of the RNAs transcribed from the human genome.^1^ The vast majority of the annotated ncRNAs have been discovered only in the past 10 years and are still largely uncharacterized (1–3). Once considered a byproduct of transcriptional noise, ncRNAs possess instead critical regulatory functions in gene expression.^1^, ^2^, ^3^ Among ncRNAs, long non‐coding RNAs (lncRNAs) are arbitrarily classified as >200 nucleotides long species, account for most of this pervasive transcription,^1^, ^2^, ^3^ and are strongly cell‐ and tissue‐restricted although not well evolutionarily conserved.^1^, ^3^, ^4^ LncRNAs exert their roles in many cellular functions operating through different mechanisms. Their versatility mainly depends on their subcellular localization and the adoption of various structural modules with interacting partners.^2^, ^5^ By regulating gene expression at epigenetic, transcriptional, and post‐transcriptional levels, lncRNAs are involved in fundamental cellular processes such as proliferation and apoptosis, development and differentiation, as well as human diseases, including cancer.^6^

Chronic Lymphocytic Leukemia (CLL) accounts for 30% of all cases of leukemia and is the most common form in adults in Western countries.^7^ CLL is characterized by the proliferation and accumulation of CD5‐positive B‐lymphocytes in the peripheral blood (PB), bone marrow (BM), lymph nodes (LN), and spleen.^8^ Clinically, CLL occurs in two forms: indolent, characterized by mutated immunoglobulin heavy chain (IGHV), and aggressive identified by unmutated IGHV genes.^9^, ^10^ Additionally, more than 80% of CLL cases present either genomic aberrations or mutations in select genes (e.g., *NOTCH1*, *TP53*, *BCL2*, and *ATM*).^11^

Few studies have investigated the role of lncRNAs in CLL (reviewed in Ref.^12^). A recent report revealed that the lncRNA treRNA is overexpressed in CLL and is associated with poor prognosis.^13^ Similarly, Croce and coworkers reported a strong correlation between lncRNA MIAT expression and disease aggressiveness since lncRNA upregulation protects CLL lymphocytes from apoptosis, ultimately sustaining monoclonal malignant B cell proliferation.^14^ Conversely, the expression of lincRNA‐p21 is significantly reduced in CLL patients, and low expression of this lncRNA demarcates a more aggressive form of CLL with a poor prognosis.^15^ LincRNA‐p21 and the paraspeckle‐enriched lncRNA NEAT1 are involved in the induction of cell death after DNA damage^16^; however, we recently observed that the expression of NEAT1 is quite heterogeneous in B cells derived from CLL patients irrespectively of cytogenetic groups or clinical outcome.^17^

LINC00152, also known as CYTOR (cytoskeleton regulator RNA), is an intergenic lncRNA initially reported to control cell cycle progression through the interaction with a network of proteins associated with the M phase of the cell cycle, thus acting as a non‐coding oncogene.^18^ Indeed, LINC00152 is significantly overexpressed in the vast majority of human cancers according to the TCGA datasets (http://ualcan.path.uab.edu/cgi‐bin/Pan‐cancer‐lncRNA.pl?genenam=CYTOR), and several studies demonstrated that its overexpression facilitates tumor progression and invasion (reviewed in Ref.^19^). On this basis, it has been proposed that LINC00152 may represent an effective target for diagnostic, prognostic, and therapeutic purposes in human cancers.

The main aim of this study was to investigate the LINC00152 expression in normal and CLL B cells in response to immunomodulatory stimuli. As an ancillary purpose, the potential prognostic role of LINC00152 was tested in a prospective cohort of newly diagnosed early stage Binet A CLL patients.

## 2 MATERIALS AND METHODS

### 2.1 Isolation of B cell subpopulations

Peripheral blood lymphocytes (PBL) B cells were negatively selected by B cells Isolation Kit II (Miltenyi cat# 130‐091‐151). B cell subpopulations were isolated by FACS sorting (FACS ARIA II, Becton Dickinson) as reported previously^20^ by staining the cells with anti‐sIgD (FITC), anti‐sIgM (PE), anti‐CD27 (PECy5), and anti‐CD19 (APC‐Vio770). The sorted B cell subpopulations were identified as reported in Table S1.

Spleen and tonsil cell suspensions were obtained using a gentle MACS dissociator (Miltenyi Biotech GmbH, Bergisch Gladbach, Germany) and mononuclear cells separated by density gradient separation Ficoll Hypaque (Seromed; Biochrom KG, Berlin, Germany).

For B‐cell subpopulations cell sorting, the following combinations of mAbs was employed: anti‐sIgD (Alexa 488; Biolegend, San Diego CA), anti‐CD19‐APC‐Vio770 (Milteni Biotech), anti‐sIgM‐PE (Dako), anti‐CD38 (PC7, BD), anti‐CD27 (PECF594, BD), anti‐CD24 (PC5, BD). Only naïve B cells, IgM memory (M‐Mem), and switched‐memory (S‐Mem) B cells were included in this study. In tonsils, memory B cells (M‐Mem) were isolated as IgD‐low/CD38^−^CD27^+^, thus comprising IgM+ and switched memory B cells. Moreover, germinal center (GC) B cells were isolated as IgD^−^/CD38^+^/CD24^−^ B cells.

### 2.2 Separation and stimulation of normal peripheral blood B cells

For *in vitro* activation with CD40L, enriched PBL B cells were cultured in the presence of a stable CD40L‐expressing NIH‐3T3 (CD40L‐TC) murine fibroblast cell line or a control NIH‐3T3 cell line stably transfected with the pIRES vector alone (Mock), at the conditions reported in Ref.^21^

To assess BCR signaling, PBL B cells were cultured with Dynabeads M‐450 Epoxy (Invitrogen, ThermoFisher Scientific) coated with (10 μg/10^7^ beads) goat anti‐Human IgM, IgD, IgG, or IgA chain specific (Southern Biotechnology, Birmingham, AL) and IL‐4 25 ng/mL (Gibco, Thermo Fisher Scientific) as previously described.^22^

As third stimulation, PBL B cells were cultured in the presence of 2.5 μg/mL CpG (ODN 2006 InvivoGen cat # tlrl‐2006‐1) and 10 ng/mL Recombinant Human IL15 (Peprotech cat# 200‐15).

### 2.3 CLL patients and CLL cell preparation

Newly diagnosed CLL patients from participating Institutions were enrolled within 12 months from diagnosis (O‐CLL1 protocol, clinicaltrial.gov identifier NCT00917540). The participants provided written informed consent in accordance with the declaration of Helsinki and the study was approved by the local institutional review board (Comitato Etico Regionale Liguria).

The diagnosis was confirmed by flow cytometry analysis together with the determination of CD38 and ZAP‐70 expression, IGHV mutational status, and cytogenetic abnormalities as previously described.^23^ TP53 and NOTCH1 mutational status was determined by PCR as previously described.^24^, ^25^

PBMCs from patients with CLL were isolated as previously reported.^26^ In all instances, the percentage of purified B cells (CD19+) exceeded 95%.

CLL B cells were stimulated like normal B cells for 48h with CD40L‐expressing NIH‐3T3 (CD40L‐TC) murine fibroblast cell line, Ig beads+IL4, and CpG+IL15.

### 2.4 Expression and silencing of LINC00152

Total RNA was isolated using the TriPure reagent (Roche) and retro‐transcribed (50 ng) using Transcriptor Reverse Transcriptase (Roche) and random hexamers according to the manufacturer's instructions. Quantitative PCR was performed using the Precision 2X QPCR master mix (Primer Design) and the Realplex II Mastercycler (Eppendorf) according to the manufacturer's instructions. Both LINC00152‐specific primers (forward: 5′‐TTC ACA GCA CAG TTC CTG GG‐3′ and reverse 5′‐GGG GGC TGA GTC GTG ATT TT‐3′) and housekeeping U6 primers (forward: 5′‐CTC GCT TCG GCA GCA CA and reverse: 5′‐AAC GCT TCA CGA ATT TGC GT‐3′) utilized for PCR reactions have been synthesized by TIB Molbiol (Genoa, Italy). OSU‐CLL cell line was obtained from the Ohio State University and previously described.^27^ MEC1 cell line was previously described.^28^ siRNA designed to knock down LINC00152 was 5′‐CUAUGUGUCUUAAUCCCUUtt‐3′ (Ambion). Control silencing was performed using the commercial control siRNA‐A (Santa Cruz, # sc‐37007). Transfections were carried out for 48 h using Kit V for Nucleofector II (Amaxa) according to the manufacturer's instructions.

### 2.5 Statistical analysis

The student's *t*‐test was performed for statistical analysis (GraphPad Prism v.6). Mann‐Whitney unpaired *t*‐test was used for non‐parametric comparisons of data sets (*p < 0.05; **p < 0.01; ***p < 0.001; ****p < 0.0001). The predictive value of LINC00152 as continuous value for discriminating patients who needed therapy from those who did not was investigated by the ROC curve analysis. An area under the ROC curve close to 0.5 indicates the complete lack of prognostic value of LINC00152. Time to First Treatment (TTFT) and overall survival (OS) analyses were performed using the Kaplan–Meier method. Statistical significance of associations between LINC00152 and TTFT or OS was calculated using the log‐rank test.

## 3 RESULTS

### 3.1 Expression of LINC00152 in normal B cell subpopulations

**FIGURE 1:**
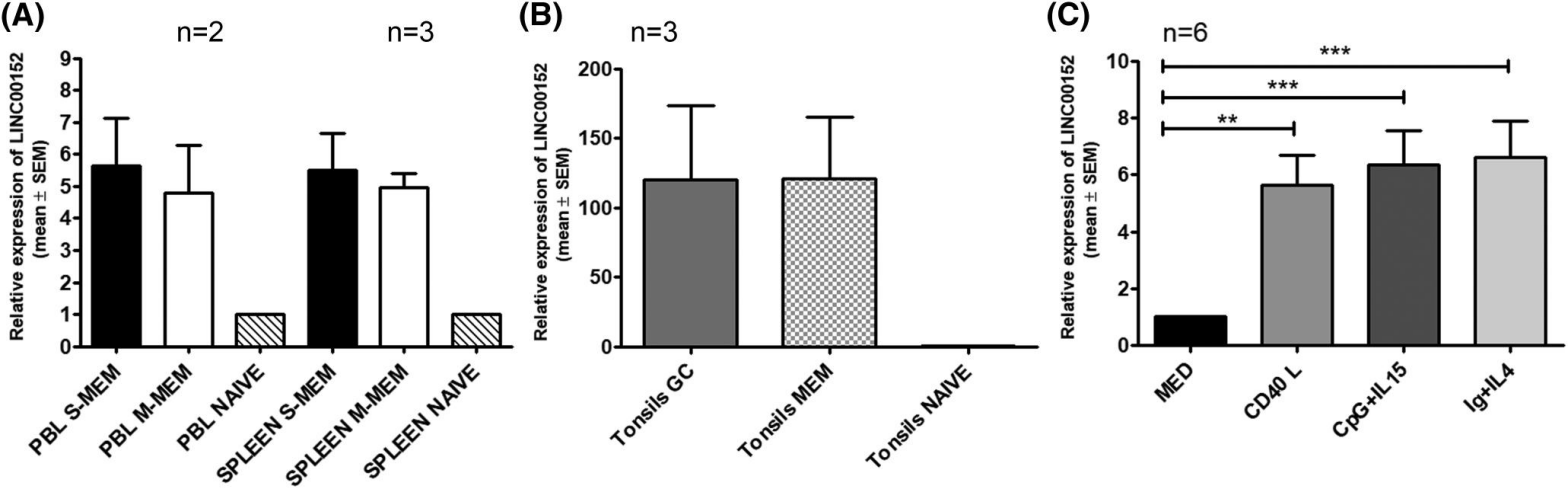
Relative expression of LINC00152 in normal B cell subpopulations derived from peripheral blood and spleen (A) and tonsils (B, tonsil samples are reported separately because of their higher expression compared to naïve B cells). (C) Relative expression of LINC00152 in purified PB B cells (*n* = 6) upon 48 h of stimulation with CD40L, CpG/IL15, and IG/IL4. **p = 0.083, ***p = 0.0006

To evaluate LINC00152 expression within normal B cells, we analyzed B cell subpopulations derived from PB (*n* = 2), spleens (*n* = 3), and tonsils (*n* = 3). Within these tissues, naïve B‐cells were identified as IgD^++^/IgM^+^/CD27^−^/CD38^−^/, IgM memory (M‐mem) B cells were isolated as IgD^+^/IgM^+^/CD27^+^/CD38^−^ and switched memory (S‐mem) B cells were isolated as IgM^−^/IgD^−^/CD27^+^/CD38^−^. In tonsils, germinal center (GC) B cells were isolated, and the memory compartments (IgM and IG switched B cells) were taken together and defined as Mem B‐cells. Strikingly, in all tissues investigated, the compartments composed of memory B cells showed significantly higher expression of LINC00152 compared to naïve B cells (Figure 1A,B). This phenomenon was most evident in B cell subpopulations derived from tonsils, where GC B cells displayed a level of LINC00152 expression similar to Mem B‐cells (Figure 1B).

### 3.2 Expression of LINC00152 is induced upon stimulation in normal B cells

To assess whether immune‐modulatory signals could modify the LINC00152 levels in normal B cells, we evaluated LINC00152 expression variations in unfractionated normal B lymphocytes. B cells were challenged with antigen‐dependent and independent stimuli (i.e., Ig/IL4 and CpG/IL15) and a T‐cell‐dependent stimulus (CD40L). After preliminary experiments, the assessment of LINC00152 expression was set at 48 h. Overall, a significant elevation of LINC00152 levels was observed after stimulation. The used stimuli were similar in inducing a substantial increase of LINC00152, with no significant differences observed among the three stimuli (Figure 1C).

### 3.3 Expression of LINC00152 in CLL cell clones

**FIGURE 2:**
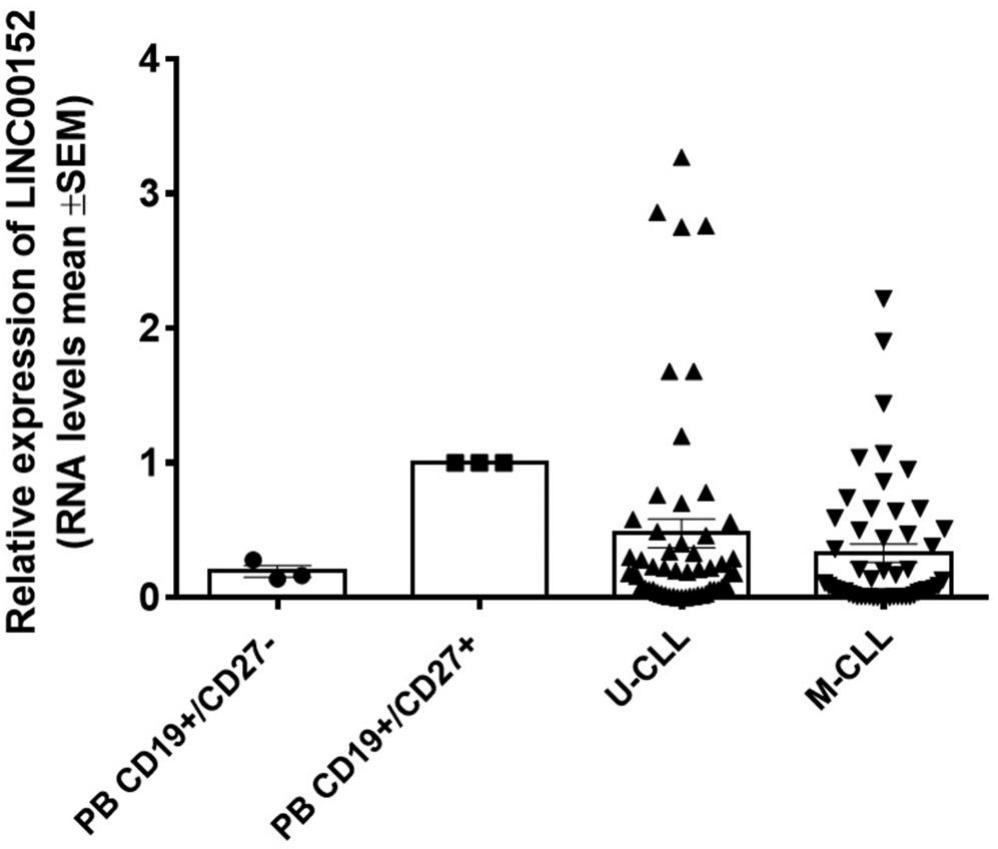
Box plot representing the LINC00152 relative expression in B cells purified from peripheral blood and in purified CLL clones. The first two bars identify three samples of naïve (CD19+/CD27‐) and memory (CD19+/CD27+) PB‐B cells; the data of IGHV U‐CLL and IGHV M‐CLL were collected from 54 and 53 cases respectively

Several lines of evidence indicate that CLL clones have the features of memory B cells.^28^, ^29^ Based on this notion, we evaluated the expression of LINC00152 in 107 purified CLL clones collected from a cohort of newly diagnosed Binet stage A patients (O‐CLL1 protocol). The expression of LINC00152 in CLL clones was compared with CD19^+^/CD27^−^ (naïve) and CD19^+^/CD27^+^ (memory) B cells derived from PB. As shown in Figures 2 and S1, the expression of LINC00152 was highly variable among CLL samples and ranged from CLL clones in which LINC00152 expression was higher than in memory B cells to CLL clones in which LINC00152 expression was lower than in naïve B cells. The latter were the majority of the patients studied. Variability of LINC00152 expression was observed both in IGHV‐mutated (M)‐ and in IGHV‐unmutated (U)‐CLL. Although U‐CLL clones averaged a higher expression level of LINC00152 than M‐CLL, differences were not statistically significant (see Figure S2).

### 3.4 Expression of LINC00152 does not predict Time to First Treatment (TTFT)

**FIGURE 3:**
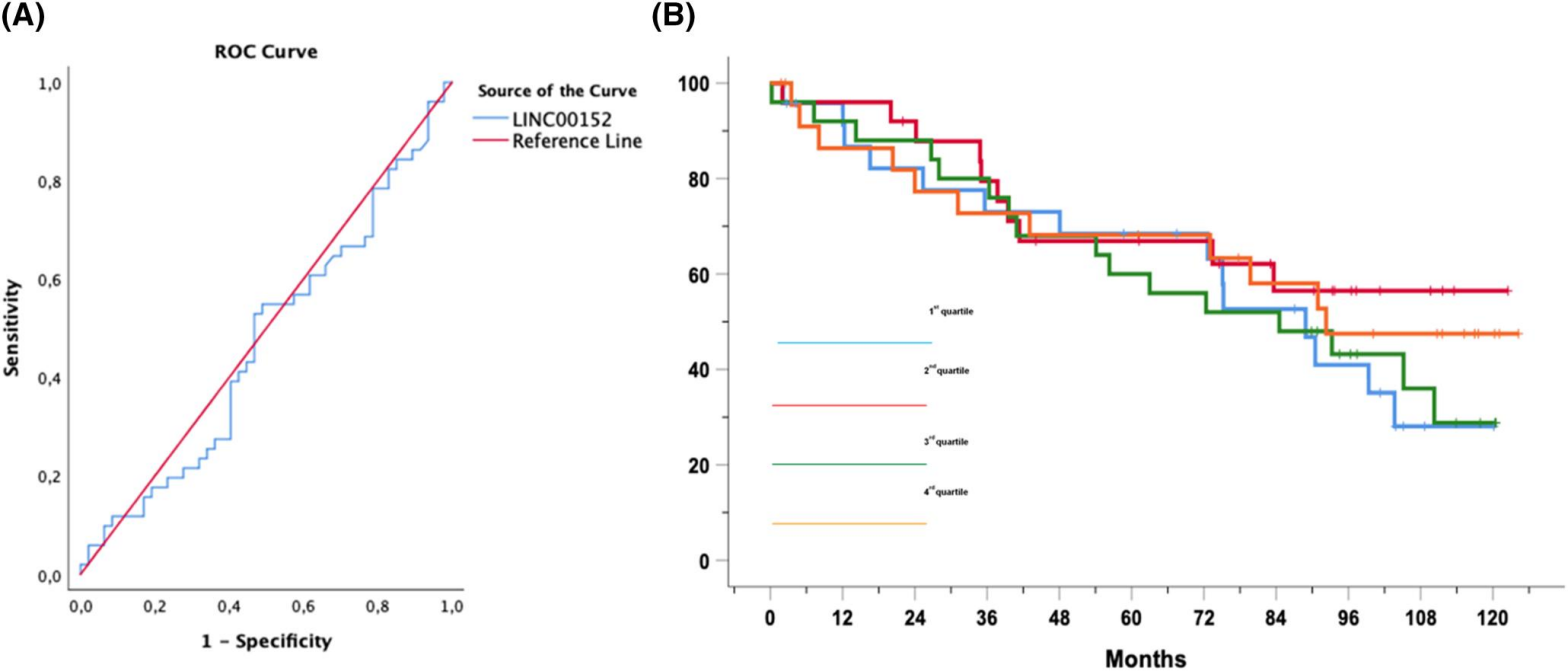
(A) Receiver Operating Characteristic (ROC) analysis of LINC00152 to identify patients who progressed. The red line represents the reference line of prognostic usefulness. (B) Kaplan‐Meier curves of the x years TTFT probability of CLL cases (*n* = 98) grouped in quartiles depending on the expression of LINC00152

Next, we investigated whether the different amounts of LINC00152 could predict TTFT of CLL patients. To this end, the clinical information of 98 patients was collected. The median follow‐up time was 75 months, and 51 patients had progressed and required therapy. LINC00152 expression largely failed to discriminate patients who needed treatment from those who did not in a Receiver Operating Characteristic (ROC) curve analysis (Figure 3A). Moreover, cases were divided into quartiles based on the expression of LINC00152 and TTFT was analyzed in the groups compared. As expected, Kaplan‐Meier estimates did not evidence statistically significant TTFT differences across LNC00152 quartiles (Figure 3B). Similarly, no OS correlation with LNC00152 expression was observed in this cohort of patients (not shown).

Further analyses were performed to evaluate whether the expression of LINC00152 could correlate with other risk factors. None of those analyzed (expression of CD38, presence of 11q‐, 13q‐, and trisomy 12, NOTCH1 mutations, and TP53 alterations) showed a significant association with LINC00152 expression levels (not shown).

### 3.5 Induction of LINC00152 expression in response to different stimuli in CLL cells

As LINC00152 could be involved in several biological cell functions in normal and neoplastic B cells, we addressed whether LNC00152 could be modulated in CLL clones as observed in normal B cells.

**FIGURE 4:**
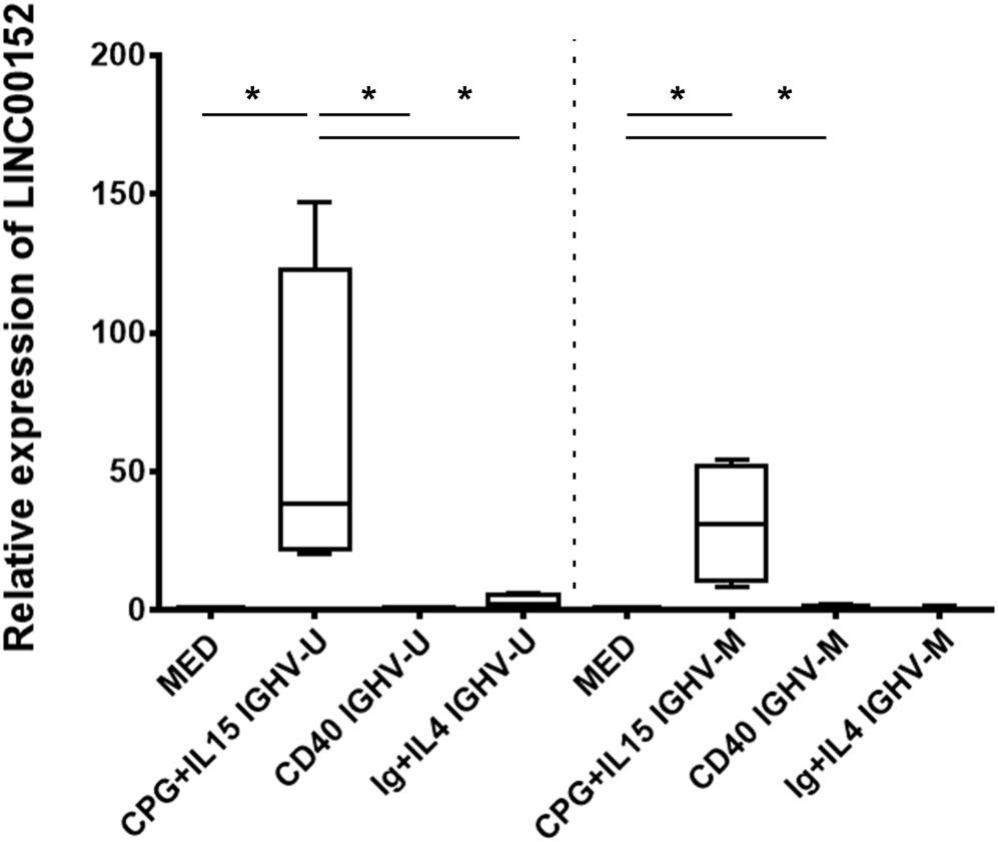
Assessment of LINC00152 expression in CLL upon several types of stimulations. Data were obtained at 48 h. In the U‐CLL significant differences were observed between medium and CpG/IL 15 stimulation (*p* = 0.0211) and between CpG/IL15 and CD40L (*p* = 0.0286) stimulations and between CpG/IL15 and Ig/IL4 stimulations (*p* = 0.0286). Among the M‐CLL, significant differences were observed between medium and CpG/IL15 stimulation (*p* = 0.0211) and medium and CD40L stimulation (*p* = 0.0319)

LINC00152 expression was evaluated in purified CLL clones stimulated with CpG/IL15, IgM/IL4, or CD40L. Similar to what was observed in normal B cells, preliminary experiments identified 48 h as the best time point to assess LINC00152 expression. LINC00152 levels were measured in eight CLL samples (4 U‐CLL and 4 M‐CLL) treated with different stimuli. As shown in Figure 4, LINC00152 was highly induced by CpG/IL15 in both M‐CLL and U‐CLL. On the contrary, expression of LINC00152 was scarcely induced in CLL cells upon CD40L and sIg/IL4 stimulations (data are summarized in Figure S3 and Table S2). However, in the M‐CLL group, CD40L/IL4 stimulation more effectively induced the expression of LINC00152.

### 3.6 Silencing LINC00152 does not induce cell death in CLL cell line(s)

**FIGURE 5:**
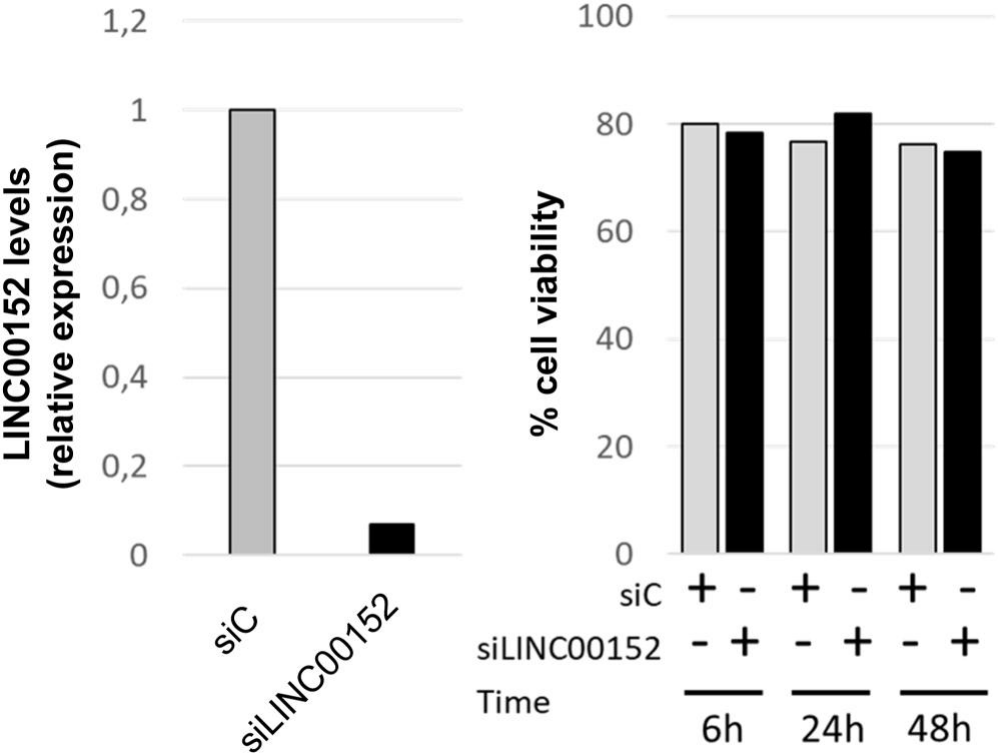
In the left panel, a representative experiment (of two) shows the OSU‐CLL cell line survival upon treatment siRNA LINC00152 (siLinc) and the control siRNA (siC). The right panel shows the silencing of LINC0152 obtained after 6 h of transfection with siRNA LINC00152 and the control siRNA

OSU‐CLL cell line, derived from the EBV infection of a leukemic clone of a CLL patient,^27^ was used to evaluate whether LINC00152 was relevant in CLL cell survival. OSU‐CLL cell line was found to express a high level of LINC00152 (not shown). We used LINC00152‐specific siRNA (and the relative control siRNA) to test whether LINC00152 could modify cell line viability in cell culture. Cultures were evaluated for cell viability and apoptosis at two different time points after siRNA transfection (24 and 48 h). Under these experimental conditions, despite the specific down‐regulation of LINC00152, we could not observe any relevant changes in cell viability and apoptosis compared with controls (Figure 5). Similar results were obtained using the cell line MEC1, derived from a CLL clone (Figure S4).

## 4 DISCUSSION

LINC00152 has been reported to be abnormally expressed in several types of human cancers (reviewed in Ref.^19^). In most cases, this information is related to solid cancers, while fewer reports present data regarding the expression of LINC00152 in hematological malignancies.^29^, ^30^, ^31^ In addition, to our knowledge, no information is available as to the role and expression of LINC00152 in normal lymphocytes.

We first addressed the expression of LINC00152 in B cell subpopulations derived from peripheral blood, tonsils, and spleens of normal donors. In addition, the expression of LINC00152 was investigated in leukemic clones derived from a cohort of newly diagnosed CLL patients.

A significantly higher expression of LINC00152 was consistently observed in memory B cell compartments in all tissues analyzed. It is believed that B‐cell memory compartments are sustained by antigen‐dependent and ‐independent stimuli,^32^, ^33^ which allow maintenance of the B‐cell memory pool. Indeed, we observed that upon stimulation of PB B cells with sIg, CD40L, and CpG, LINC00152 was significantly upregulated, suggesting its involvement in the molecular mechanisms that allow B cell memory long‐term survival. The possibility that naïve and memory B cell compartments have different abilities to regulate LINC00152 expression has to be considered, and it will be investigated in forthcoming studies.

Because of this expression pattern, we decided to investigate LINC00152 expression in ex vivo CLL cell clones. In a cohort of 107 early stage Binet A patients collected at diagnosis,^23^ the expression of LINC00152 was highly heterogeneous. It ranged from levels higher than those detected in memory PB B cells to levels markedly lower than those detected in naïve PB B cells. As an increased expression of LINC00152 has been linked to features of aggressiveness in other tumor types,^19^ we correlated LINC00152 expression with several molecular markers of CLL clones capable of predicting disease evolution (i.e., IGHV mutational status, CD38 expression, alterations of chromosomal asset, and modifications of *TP53*). No association was found between LINC00152 expression and known prognostic markers. In addition, we failed to demonstrate any possible correlation of LINC00152 expression with clinical outcomes expressed by TTFT and OS.

In CLL cells, stimulation via Toll‐like Receptor 9 using unmethylated CpG oligodeoxynucleotides in association with IL15 significantly upregulated LINC00152. On the contrary, the stimulation with CD40L and sIg generally provided a limited modulation of the LINC00152 level in CLL cells (Figures 4 and S3). This is markedly different from what we observed in normal B cells, where sIg, CD40L, and CpG stimulations were similarly efficient in enhancing LINC00152 expression. sIg deficient signaling observed in CLL clones, due to their state of relative anergy^34^, ^35^, may justify this result. As for CD40L stimulation, a degree of heterogeneity in the capacity of CLL cells to respond through CD40 molecule^36^, ^37^ has been reported. In addition, although T‐cells are believed to be indispensable for CLL engraftment of immunodeficient mice,^38^ a recent study in a murine model of CLL indicates that this might not be dependent on CD40‐CD40L interaction.^39^ These results are in keeping with the notion that CLL cells show a certain degree of functional derangement (reviewed in Ref.^40^). Altogether these results reinforce the idea that the level of LINC00152 is mainly dictated by immunomodulatory signals rather than by the “status” of naïve or memory B cells. A more accurate evaluation of possible differences between U‐ and M‐CLL in the ability to modulate LINC00152 expression requires further studies, including testing a higher number of U‐ and M‐CLL cases.

Finally, we investigated whether LINC00152 silencing was capable of modifying CLL cell viability. This experiment was performed in CLL‐derived cell lines (OSU‐CLL and MEC1) as these cells constitutively have a high expression of LINC00152 compared to most CLL clones (not shown). Upon efficient silencing of LINC00152, we could not identify significant cell viability changes and apoptosis induction (Figures 5 and S4).

Thus, though LINC00152 expression appears to modulate tumor cell survival in other cancer models, its role in CLL cell survival and expansion remains to be determined. It can be speculated that the lack of TTFT‐ and OS‐prognostic value of LINC00152 expression in CLL could be ascribed to the fact that the leukemic cells derived from the PB. Since CLL disease might be promoted mainly by microenvironment stimuli in the lymphoid tissues, LINC00152 importance may be dictated by its modulation following stimuli occurring in the lymphoid microenvironment. In this vision, CpG/IL15 would be the best stimulus capable of inducing LINC00152 expression.

Altogether, our observations do not support a role for LINC00152 in CLL progression. However, it cannot be ruled out the possibility that LINC00152 may exert some role in supporting leukemic cell evolution due to its possible regulation in the lymphoid microenvironment in more advanced stages of the diseases. This latter point should be addressed by studying a cohort of progressed or relapsed patients.

Overall, this is the first study describing the preferential expression of LINC00152 in the memory B cell compartment. In addition, we show that LINC00152 can be induced in normal B cells and CLL clones upon different immunomodulatory signals, of which only CpG/IL15 appears to be effective in CLL cells. In both normal and leukemic B cells, the role of this regulatory lncRNA needs further investigation.

## CONFLICT OF INTEREST

The authors have no conflict of interest to disclose.

### PEER REVIEW

The peer review history for this article is available at https://publons.com/publon/10.1002/hon.2938.

## Supporting information

Supporting Information S1

## Data Availability

The data not reported in the article that supports the findings of this study are available from the corresponding author upon reasonable request.
