# Supplementary material for: LINC00152 expression in normal and Chronic Lymphocytic Leukemia B cells
Source: Hematol Oncol. 2021 Oct 28;40(1):41–8. doi: 10.1002/hon.2938 (PMC9297877; doi:10.1002/hon.2938)
Supplement: Supplementary file 1 — Supporting Information S1 [file HON-40-41-s001.docx]

**Supplementary files**


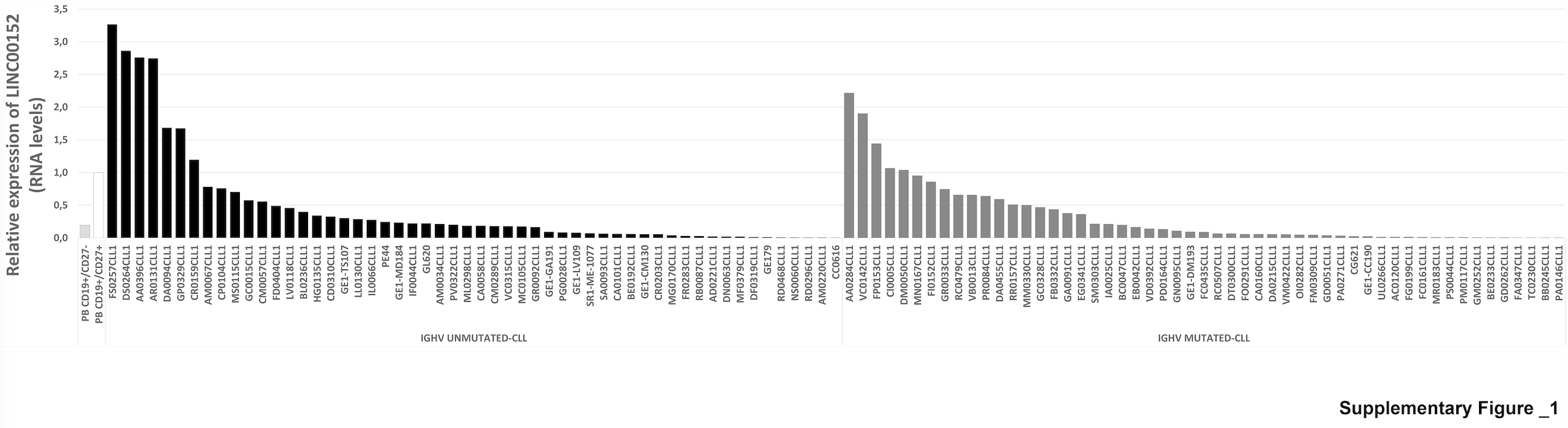


**Supplementary figure 1.** LINC00152 relative expression of B cells purified from peripheral blood compared to what observed in purified CLL clones. The first two bars identify RNA samples derived from 3 samples of naïve (CD19+/CD27+) and memory (CD19+/CD27+) PB-B cells; black histograms identify U-CLL clones; grey histograms identify M-CLL clones.


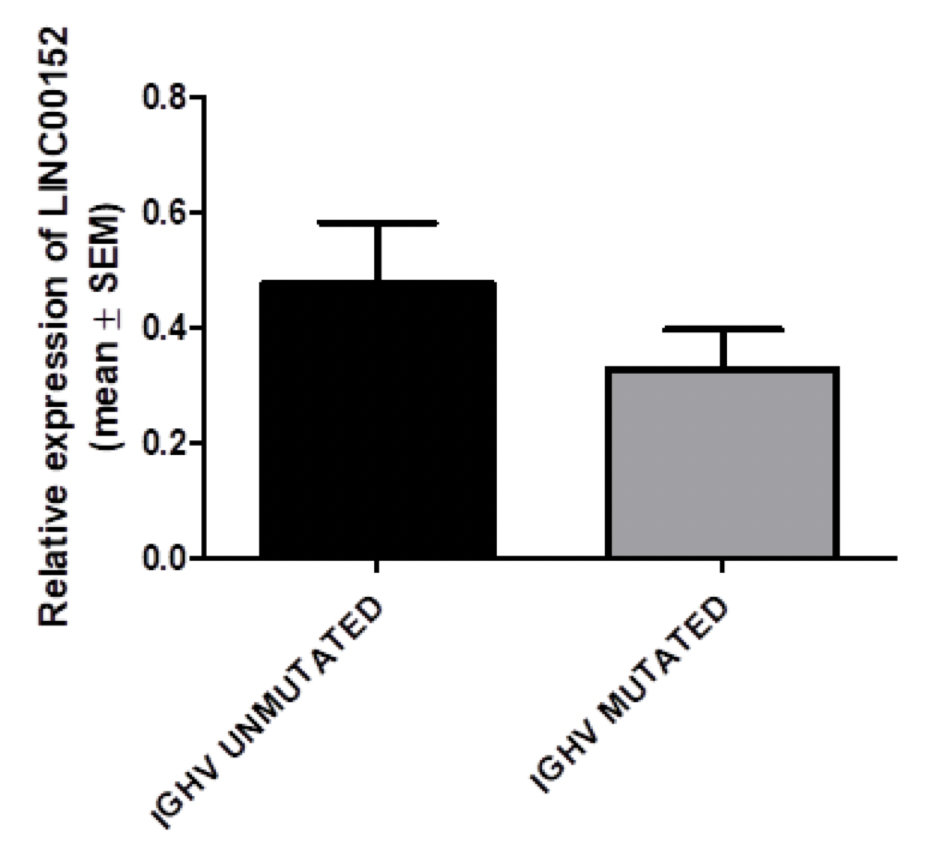


**Supplementary figure 2.** Comparison of LINC00152 expression between U-CLL (n=54) and M-CLL (n=53) clones. Differences were not statistically significant (p=0,225).


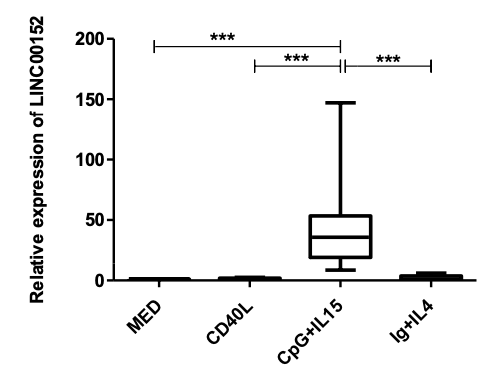


**Supplementary figure 3.** Cumulative data showing the assessment of LINC00152 expression in CLL upon several types of stimulations. Measures were obtained at 48 hours.


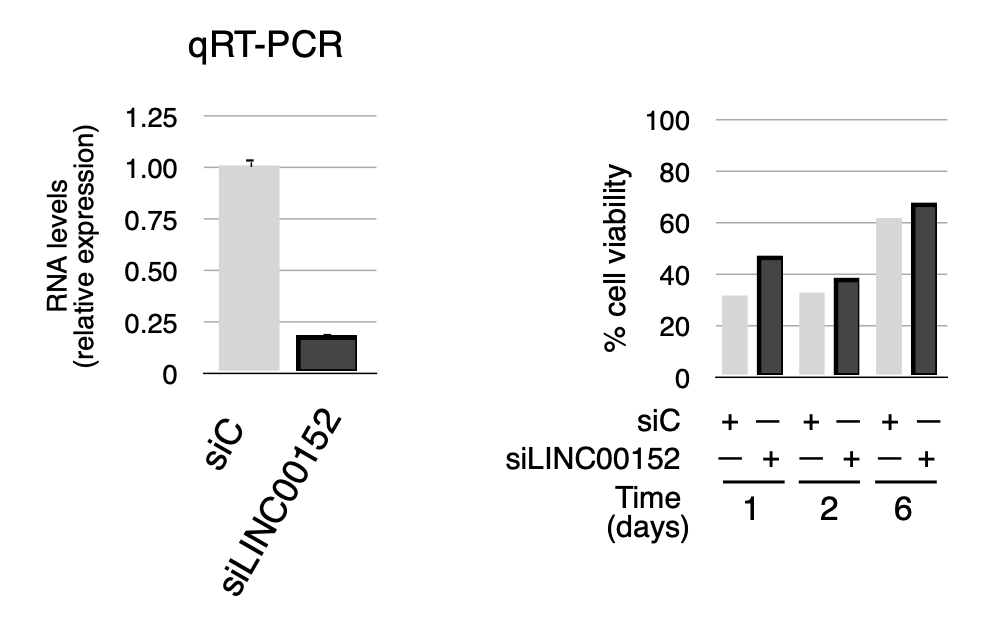


**Supplementary figure 4**. In the left panel, a representative experiment shows the silencing of LINC0152 obtained after 6 hours of transfection with siRNA LINC00152 (siLINC00152, black bar) and the control siRNA (SiC, grey bar) obtained in the MEC1 cell line. The right panel shows the cell line survival at several time-points upon treatment siRNA LINC00152 and the control siRNA.

|  | **Surface phenotypes** | | | | | |
| --- | --- | --- | --- | --- | --- | --- |
| **Tissues** | *Naive* | *Germinal Center* | *IgM-mem* | *Switch- memory* |  |  |
| PB | IgD++, IgM+, CD19+, CD27- |  | IgD-low, IgM+, CD27+ | IgD-low, IgM-, CD27+ | |  |
|  |  |  |  |  | |  |
| Tonsils | IgD++, CD38-, CD27-, IgM+ | IgD-, CD24-, CD38+ | Memory: IgD-low, CD38-, CD27+ | | | |
|  |  |  |  |  | |  |
| Spleens | IgD++, CD38-, IgM+, CD27- |  | IgD-low, CD38-, IgM+, CD27+ | IgD-low, CD38-, IgM-, CD27+ | |  |

**Supplementary table 1.** Phenotype of the B-cell subpopulations sorted from peripheral blood (PB), tonsils and spleens.

|  | **CLL samples** | **MEDIUM** | **CD40L** | **CpG+IL15** | **αIgM+IgD+IL4** |
| --- | --- | --- | --- | --- | --- |
| **IgHV UNMUTATED** | GE179 | 1 | 1,38 | 147,03 | 6,06 |
|  | GE1-LV109 | 1 | 0,88 | 49,7 | 1,15 |
|  | GE1-TS107 | 1 | 0,92 | 20,49 | 3,8 |
|  | GE1-CM130 | 1 | 1,02 | 27,3 | 0,37 |
| **IgHV MUTATED** | CG621* | 1 | 2,54 | 8,6 | 0,44 |
|  | GD0262CLL1 | 1 | 1,88 | 54,55 | 0,52 |
|  | PS0080CLL1 | 1 | 1,46 | 18,63 | 0,81 |
|  | GE1-DM193 | 1 | nd | 44,32 | 1,89 |

*** IgG CLL**

**Supplementary table 2.** The numbers indicate the relative expression of LINC00152 observed in CLL clones upon the challenge with the indicated stimuli at 48 hours.

nd= not done
